# Assessment of PD-L1 mRNA expression in gastrointestinal tumors and the response to immunotherapy

**DOI:** 10.3389/fonc.2022.926746

**Published:** 2022-12-01

**Authors:** Qingqing Qiu, Jieyi Li, Qiaofeng Chen, Xiaokai Zhao, Ru Zhou, Wenpeng Zhang, Ziying Gong, Daoyun Zhang, Mingliang Wang

**Affiliations:** ^1^ Department of General Surgery, RuiJin Hospital/Lu Wan Branch, School of Medicine, Shanghai Jiaotong University, Shanghai, China; ^2^ Jiaxing Key Laboratory of Precision Medicine and Companion Diagnostics, Jiaxing Yunying Medical Inspection Co., Ltd., Jiaxing, Zhejiang, China; ^3^ Department of R&D, Zhejiang Yunying Medical Technology Co., Ltd., Jiaxing, Zhejiang, China; ^4^ Department of General Surgery, Ruijin Hospital, School of Medicine, Shanghai Jiaotong University, Shanghai, China

**Keywords:** PD-L1, IHC, qRT-PCR, consistency, gastrointestinal tumors

## Abstract

**Background:**

Programmed death ligand 1 (PD-L1) immunohistochemistry (IHC) has been proposed as a predictive biomarker to predict response to immunotherapy. Given the limitations of IHC test in PD-L1 detection, this study aimed to investigate the technical feasibility of using quantitative RT-PCR (qRT-PCR) to replace IHC in PD-L1 detection in gastrointestinal tumors.

**Materials and methods:**

The Cancer Genome Atlas database was used to evaluate the relationship between PD-L1 expression in tumor tissue and the patient prognosis. In addition, 52 patients with gastrointestinal cancer were enrolled and divided into the stomach (STAD), colon (COAD), and rectum (READ) adenocarcinoma cohorts. IHC test was used to determine the PD-L1 level of the tissue specimens, and the qRT-PCR test was used to analyze the mRNA expression in both blood and tissue specimens. Moreover, the correlation between blood PD-L1 mRNA expression and immunotherapy efficacy was investigated in additional 15 patients with gastric cancer that further enrolled.

**Results:**

The expression level of PD-L1 in tumor tissue is related to the tumor stage of COAD (*p-value* = 0.001) and primary therapy outcomes in patients with READ (*p-value* = 0.003) but not significantly correlated to the overall survival (OS) time of patients with gastrointestinal cancer. Moreover, the concordance of PD-L1 mRNA expression level of tissue and paired blood samples is low, despite a weak linear relationship that was found in the STAD cohort (*r* = 0.43, *p-value* = 0.049). We further demonstrated that qRT-PCR results in both tissue and blood specimens were numerically but not statistically significant consistent with IHC results (corresponding to a *p-value* of 0.84 and 0.55, respectively). Remarkably, high PD-L1 expression in blood of patients with STAD shows a better response to immunotherapy (*p-value* = 0.04), which could be well identified at the relative expression cutoff of 1.5 (sensitivity of 85.7%, specificity of 75.0%, and AUC of 0.82).

**Conclusions:**

Our study established a novel strategy for rapidly distinguishing patients with gastrointestinal cancer with the response to immunotherapy and has potential clinical benefits.

## Introduction

Gastrointestinal tumor mainly includes gastric, colon, and rectal cancers. Gastric cancer (GC) is the most common gastrointestinal tumor in the world, and elevated expression of programmed death ligand 1 (PD-L1) has been reported in up to 65% of GC/gastroesophageal junction cancer (GEJC) and is associated with specific molecular subtypes of gastric adenocarcinoma (1). The PD-L1 was well known as an immune checkpoint marker where it binds to its receptor PD-1 expressed on activated T cells, leading to immune suppression (2). Some clinical trials involving PD-L1 inhibitors showed a consistent correlation between the patient response rates and the PD-L1 expression level of the tumor (3), displaying that, for patients with high PD-L1 expression, utilization of immunotherapy such as pembrolizumab could significantly improve the clinical outcomes (4). For example, on the basis of the promising clinical activity observed in the KEYNOTE-059 trial, pembrolizumab was approved for the treatment of patients with chemotherapy-refractory PD-L1–positive GC/GEJC cancer (5). Whereas an overall survival (OS) favoring nivolumab over placebo regardless of PD-L1 expression status was observed in the ATTRACTION-2 trial (6).

To date, the expression level of PD-L1 is mainly measured and evaluated by the immunohistochemistry (IHC) method and acts as an indicator that benefits the therapy design and becomes one of the standards of care in oncology (7). Notably, the IHC protocols used to determine PD-L1 varied considerably with different antibodies and staining cutoffs, and because of these limitations, the process should be carefully standardized (8). The blueprint project indicated that interchanging assay methods and cutoff criteria for PD-L1 positivity can lead to inconsistent classifications of PD-L1 status in some patients, highlighting the limitations of the current IHC approach to assessing PD-L1 expression in terms of reproducibility and in sampling variability (9). It is now well accepted that PD-L1 expression is heterogeneous in the tumors of most types of cancer, especially gastrointestinal tumors. A recent study showed discordance in PD-L1 assessment between biopsy and surgically resected specimens (10).

Given the limitations of PD-L1 IHC, alternative diagnostic strategies have been studied to find better predictors of response to PD1/PD-L1 blockade. Recently, studies showed that exosomes isolated from the plasma of patients with tumor express PD-L1 and play a role in immune escape by reducing T-cell activity and promoting tumor growth (11). Chen et al. (12) discovered that the level of circulating exosomal PD-L1 was associated with anti–PD-1 response in patients with metastatic melanoma. The PD-L1 mRNA expression in plasma-derived exosomes was also found associated with response to anti–PD-1 antibodies in melanoma and NSCLC (13). Fan et al. (14) found that exosomal PD-L1 was an independent prognostic factor in GC and that OS was significantly lower in the high– exosomal PD-L1 group compared with that in the low– exosomal PD-L1 group. Together, these studies demonstrate exosome PD-L1 expression is a potential biomarker for immunotherapy and prognosis. However, because of the heterogeneity in size and molecular contents of exosomes, the isolation and enrichment methodologies are time-consuming, complicated equipment-based, high-cost, and lengthy process, which limited the widespread clinical application (15, 16).

Notably, plasma cell-free RNAs could successfully quantitate the PD-L1 mRNA expression level in patients with gastric and colorectal cancer (17); predict the benefit of immunotherapy treatment of patients with lung cancer (18); and display a rapid, convenient, and effective alternative in discovering novel PD-L1 expression detection approaches. Given the contribution of expanding the understanding of the plasma PD-L1 mRNA expression and exploring its prognostic value in immunotherapy in patients with gastrointestinal tumor, the present study investigated the correlation of PD-L1 mRNA expression in blood and paired tumor tissue specimens, compared the consistency of IHC and quantitative RT-PCR (qRT-PCR) test, and further inquired about the relationship between plasma PD-L1 mRNA expression level and the response to the immunotherapy.

## Materials and methods

### TCGA database screening

MEXPRESS (https://mexpress.be/index.html) (19) was used for screening and identifying the expression characteristics of PD-L1 in The Cancer Genome Atlas (TCGA) database of stomach adenocarcinoma (STAD), colon adenocarcinoma (COAD), and rectum adenocarcinoma (READ), respectively. Moreover, the relationship between survival time of the patient and the PD-L1 expression level was analyzed by UALCAN (http://ualcan.path.uab.edu/) (20) in TCGA database records.

### Ethics approval and consent to participate

This study was conducted according to the principles of the World Medical Association Declaration of Helsinki. The study was approved specifically by the Internal Review Board of the Ruijin Hospital Affiliated to Shanghai Jiaotong University School of Medicine. All participants provided written informed consent for this retrospective study (IRB approval no. 20190411).

### Patient characteristics

From April 2019 to April 2020, patients with suspected gastrointestinal cancer diagnosed and treated at Lu Wan Branch of Ruijin Hospital were enrolled in this study. Exclusion criteria were unwilling to fully participate in the whole study, including those diagnosed as the non-gastrointestinal tumor and uncommitted to participate in PD-L1 expression tests. Data regarding the clinical characteristics of each patient were carefully collected. Diagnosis of diseases was established using clinical and endoscopy findings and confirmed by histopathology stain. All patients included in the study provided at least 10 ml of blood specimens, and paired tissue specimens were obtained by the operation. Moreover, we obtained control blood specimens from 11 healthy people, they were clinical diagnosed and confirmed without gastrointestinal tumors, and were enrolled as a negative control cohort.

### IHC and H&E staining

Briefly, tissue specimens were paraffin-embedded and sectioned into 4 μm, and hematoxylin and eosin (H&E) staining were performed using a standard protocol. Moreover, paraffin sections quantified the expression level of PD-L1 using IHC technology and stained in one batch by 22C3 assays (Agilent, CA, USA). In addition, the percentage of tumor cells and IHC results were both evaluated retrospectively by two experienced pathologists independently and divided into < 1% and > 1% subgroups based on positively stained cells of IHC. Correspondingly, microscopic observation of tumor cells and IHC result was conducted and photographed using an optical microscope (BX43, Olympus, Tokyo, Japan).

### qRT-PCR analysis

Each process was strictly following the manufacturer’s protocols. Total RNA of tissue specimens was isolated by the RNA simple Total RNA Kit (Tiangen, Beijing, China), and the Blood RNAPlus Kit (Yunying, Shanghai, China) was used for extracting total RNA from plasma samples obtained from centrifugation of 10 ml of blood specimens. Reverse transcription was performed by the HiScript^®^ III 1st Strand cDNA Synthesis Kit (Vazyme, Nanjing, China), and qRT-PCR analysis was performed by an Alldetect™ kit (Yunying, Shanghai, China) using an ABI 7500 system (ThermoFisher, MA, USA). The TaqMan assay was used in qRT-PCR amplify detection. Primers and probes (**Supplementary Table S1**) were designed by Primer Premier 5.0 (Premier Biosoft, CA, USA) and synthesized by Generay (Generay Biotech, Shanghai, China). Average ΔCT of the negative control group was calculated (**Supplementary Table S2**), and quantification of PD-L1 expression of patients with gastrointestinal cancer was studied and estimated on the basis of the relative 2^−ΔΔCT^ method.

### Treatment schedules and regimens

A total of 15 patients who were diagnosed with STAD were further enrolled, and blood specimens were obtained and detected PD-L1 expression from them before treatment. All participants received pembrolizumab at 200 mg in combination with paclitaxel-albumin at 260 mg/m^2^ body surface area every 3 weeks. Treatment responses were assessed after three cycles, and building upon the efficacy, each participant was divided into good responder (partial response, the diameters of lesions at least decreased 30% refer to the baseline) and poor responder (progressive disease, the diameters of lesions at least increased 20% or another lesion appears; stable disease, the diameters of lesions not changed).

### Statistical analysis

One-way analysis of variance (ANOVA) was used to analyze the significance of the difference in this study, followed by Tukey’s tests that were used for IHC results and tumor cell percentage, and Pearson’s correlation coefficient test was used for the correlation analysis between PD-L1 mRNA expression and tumor cell percentage. The Bland–Altman analysis was used to compare the consistency of qRT-PCR results of different specimen types in the same patient, and the linear regression analysis was employed to inquire the potential relationship of PD-L1 mRNA expression level between blood and paired tissue specimens. Dunnett’s tests were used to independently compare the IHC results with the qRT-PCR results. All statistical analyses were conducted with SPSS 22.0 (IBM, Chicago, USA); *p-value* less than 0.05 was considered as a significance level. The receiver operating characteristic (ROC) curve was plotted by R package “pROC” (21), and boxplots were generated by R package “ggplot2” (22).

## Results

### Integrative analysis of PD-L1 expression level in TCGA database

**Figure 1:**
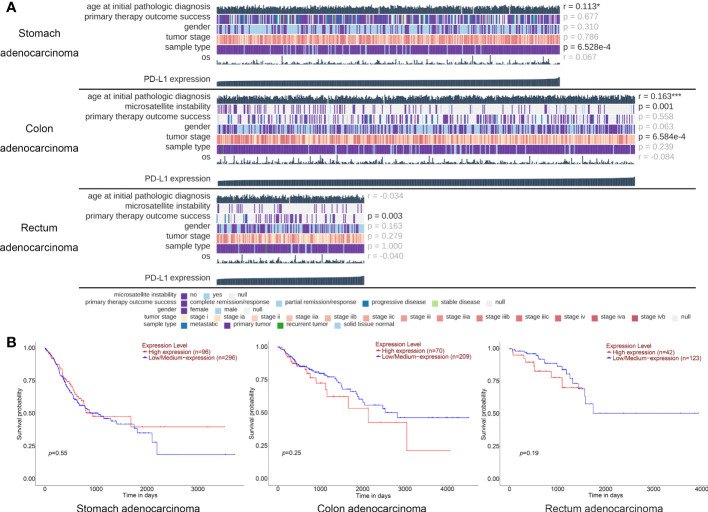
TCGA database analysis of the PD-L1 expression and baseline information for patients with gastric cancer. **(A)** The oncoplots generated on the data of PD-L1 expression and personal baseline in patients with stomach adenocarcinoma, colon adenocarcinoma, and rectum adenocarcinoma, respectively. Each image was generated and analyzed by the MEXPRESS, each column represents a sample, whereas each row represents a different clinical variable. The numbers on the right side of the figure show the Benjamini–Hochberg adjusted p-values and Pearson correlation coefficients for the comparison between the PD-L1 expression levels and all the other variables. * p < 0.05, *** p < 0.001. **(B)** The survival curve of different PD-L1 expression level in patients with stomach adenocarcinoma, colon adenocarcinoma, and rectum adenocarcinoma, respectively; each image was generated and analyzed by the UALCAN.

Followed by TCGA database screening, we first investigated the relevance between the baseline characteristics and PD-L1 expression level. A total of 407 patients with STAD, 499 patients with COAD, and 177 patients with READ with PD-L1 expression in tumor tissue were selected from TCGA database. Results indicated that the expression level of PD-L1 is less correlated to the age, gender, and OS of patients with gastrointestinal tumors. Notably, although the PD-L1 expression is highly related to tumor stage in patients with COAD (*p-value* = 0.001) and significant related to primary therapy outcomes in patients with READ (*p-value* = 0.003), no obviously correlations were found between the OS time and PD-L1 expression (**Figure 1A**).

To further demonstrated, Kaplan–Meier analysis was performed to show the relationship between PD-L1 expression level and survival probability of these patients, and we found that the expression level of PD-L1 was not significant related to the patients’ survival time of STAD (*p-value* = 0.55), COAD (*p-value* = 0.25), and READ (*p-value* = 0.19) (**Figure 1B**). These results suggested that the expression level of PD-L1 in tumor tissue of gastrointestinal cancer is not a clinical benefit prognostic indicator.

### Patient characteristics

However, the expression level of PD-L1 in tumor cells often indicates the efficacy of Pembrolizumab treatment. To examine whether PD-L1 expression level could quantify *via* qRT-PCR detection methods, a total of 52 patients newly diagnosed with gastrointestinal cancer were enrolled in this study, and the median age was 64.5 years. The tumor size and stage of each patient were carefully measured and evaluated (**Supplementary Table S3**), and the characteristics of the study participants are shown in **Table 1**. On the basis of the primary tumor and pathologic type, patients were further divided into STAD, COAD, and READ cohort. The median age of STAD, COAD, and READ cohorts was 65, 63, and 62 years, respectively. The major gender and tumor stage of STAD, COAD and READ cohort was male [corresponding to 13 (56.5%), 10 (52.6%), and 7 (70.0%), respectively] and stage III [corresponding to 8 (34.8%), 9 (47.4%), and 5 (50.0%), respectively].

**Table 1:** Patients’ characteristics.

Characteristics	All patients
	52
*Gender*	
Male	30 (57.7%)
Female	22 (42.3%)
*Age at Diagnosis in Years*	
<60	15 (28.8%)
>60	37 (71.2%)
*Clinical diagnosis*	
STAD	23 (44.2%)
COAD	19 (36.5%)
READ	10 (19.2%)
*Tumor stage*	
I	12 (23.1%)
II	12 (23.1%)
III	22 (42.3%)
IV	6 (11.5%)

STAD, stomach adenocarcinoma; COAD, colon adenocarcinoma; READ, rectum adenocarcinoma.

### IHC assay of PD-L1 protein expression

**Figure 2:**
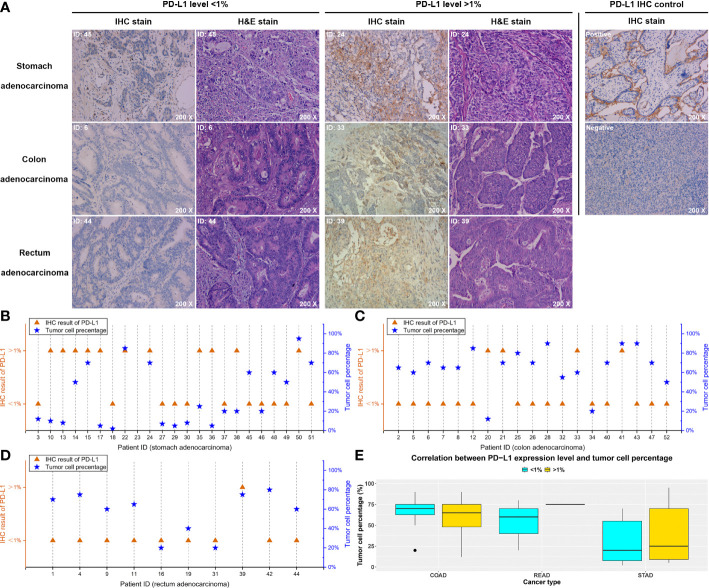
IHC test of the PD-L1 expression level and pathological staining results. On the basis of the IHC results, each patient was divided into high level (> 1%) and low level (< 1%) of PD-L1 expression. **(A)** Typical images of stomach, colon, and rectum adenocarcinoma, respectively, and the corresponding pathological results. **(B–D)** The relationship between the PD-L1 expression levels detected by IHC and the tumor cell percentages of each tissue specimen in the stomach, colon, and rectum adenocarcinoma, respectively. **(E)** The statistical analysis of the correlation between PD-L1 expression level and tumor cell percentage.

IHC assay was performed in all the 52 patients, and the PD-L1 protein expression level was quantized. On the basis of the IHC results, the PD-L1 protein expression was evaluated and divided into high (>1%) and low (<1%) levels (Fig ure 2A and **Supplementary Table S4**). For ruling out the deviations of IHC result caused by the difference of tumor cell number in tissue samples, tumor cells in each specimen were further counted and confirmed using H&E staining (**Figure 2A**). Only the samples with a result that at least 100 tumor cells were contained in each of six randomly selected microscopic fields were considered as qualified. Our data showed that, in all qualified samples, no significant relationship between the tumor cell content and the PD-L1 expression level was observed (*p-value* = 0.27), and similar results were found in STAD (*p-value* = 0.15, **Figure 2B**), COAD (*p-value* = 0.76, **Figure 2C**), and READ (*p-value* = 0.41, **Figure 2D**) subgroups (**Figure 2E**).

### qRT-PCR assay of PD-L1 mRNA expression

**Figure 3:**
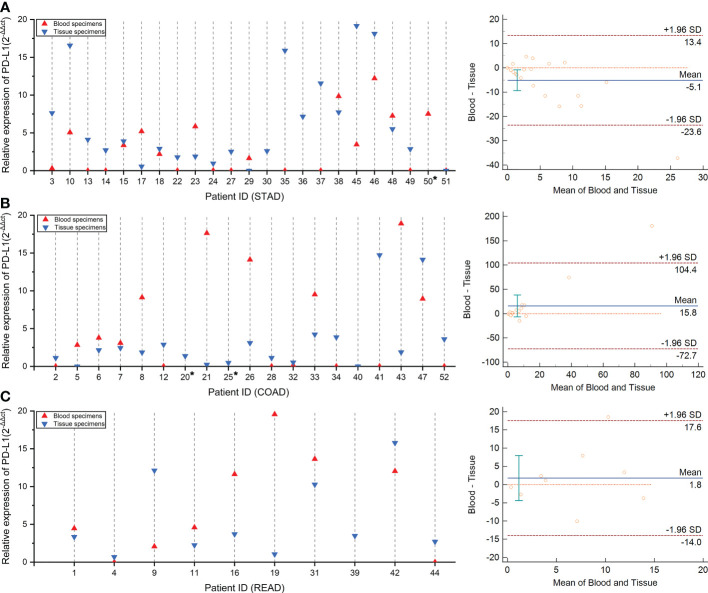
The concordance of PD-L1 mRNA expression level between tumor tissue and blood specimens *via* qRT-PCR test. Panels **(A–C)** compared the consistency of PD-L1 expression in tissue and blood specimens *via* qRT-PCR test in patients with stomach, colon, and rectum adenocarcinoma, respectively. The concordance rate and statistical analysis were performed by the Bland–Altman graph, which was drawn on the right side.

We further performed a qRT-PCR assay to quantize the mRNA expression of PD-L1 in both tissue and blood specimens (**Supplementary Table S4**). Among the 52 tested patients, mRNA expression of PD-L1 was successfully detected in each tissue specimen. However, four blood specimens failed to detect the PD-L1 mRNA expression, corresponding to two in STAD (ID 32 and ID 36, **Figure 3A**), one in COAD (ID 40, **Figure 3B**), and one in READ (ID 39, **Figure 3C**), respectively. Notably, no obvious correlations were discovered between the tumor cell percentage and PD-L1 mRNA expression in tissue specimens (r = −0.16, *p-value* = 0.27) (**Figure S1**).

Followed by the comparison of the consistency between qRT-PCR test results in the paired blood and tissue samples, we observed that the concordance rate of PD-L1 mRNA expression level of these two samples is low in the STAD, COAD, and READ cohort (**Figures 3A–C**). Remarkably, a weak linear correlation trend was demonstrated in STAD cohort (*r* = 0.43, *p-value* = 0.049, **Figure S2A**), despite no correlations were found in either COAD cohort (*r* = 0.21, *p-value* = 0.4, **Figure S2B**) or READ cohort (*r* = 0.17, *p-value* = 0.661, **Figure S2C**). These results suggested that the evaluation system of PD-L1 mRNA expression level should be independently established on the different specimen types.

### Comparison between mRNA and protein expression level of PD-L1

**Figure 4:**
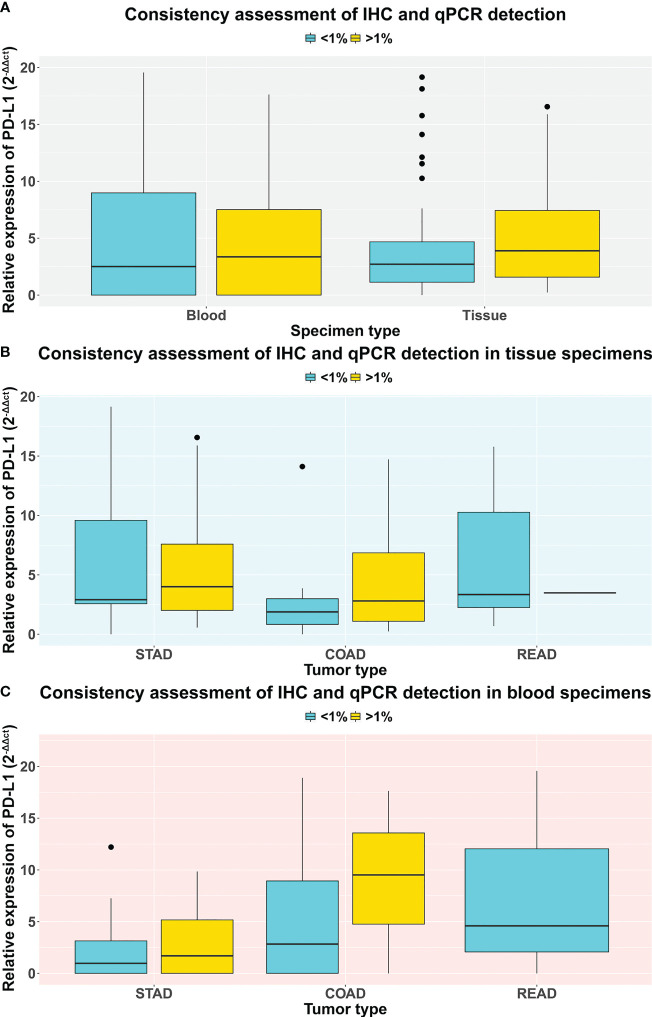
The comparison of PD-L1 expression level qualified by IHC and qRT-PCR methods. Each patient was grouped into a high (> 1%) and low (< 1%) cohort based on their IHC results of PD-L1 expression. **(A)** The comparison of PD-L1 expression level between tissue and blood specimens *via* qRT-PCR test. **(B)** The comparison of PD-L1 expression level between stomach, colon, and rectum adenocarcinoma patients *via* qRT-PCR test on tissue specimens. **(C)** The comparison of PD-L1 expression level between stomach, colon, and rectum adenocarcinoma patients *via* qRT-PCR test on blood specimens.

Referring to IHC results of PD-L1 protein expression level, we re-grouped the patients into high (>1%) and low (<1%) cohorts and then analyzed the correlation between PD-L1 mRNA expression level and protein levels. Interestingly, in both blood and tissue specimens, we found that, compared with the low cohort, mRNA expression levels showed a numerically but not statistically significant upregulated trend in the high cohort (**Figure 4A**, *p-value* = 0.84 and 0.55 in blood and tissue, respectively; **Supplementary Table S5**). We further inquired the concordance between IHC and qRT-PCR quantification results of PD-L1 expression in different tumors. Results showed that, in tissue specimens, the average level of PD-L1 mRNA expression is higher in the IHC quantification >1% group than that in <1% group, and this trend was established in both patients with STAD and COAD, although no statistical significance were found (**Figure 4B**, *p-value* = 0.86 and 0.30 in STAD and COAD, respectively; **Supplementary Table S5**). Similar results were found in blood samples (**Figure 4C**, *p-value* = 0.82 and 0.49 in STAD and COAD, respectively; **Supplementary Table S5**), indicating that the mRNA level may be able to predict the IHC determination, and further rigorous assessments in a large cohort are required.

### Correlation between PD-L1 expression level in blood and immune efficacy

**Figure 5:**
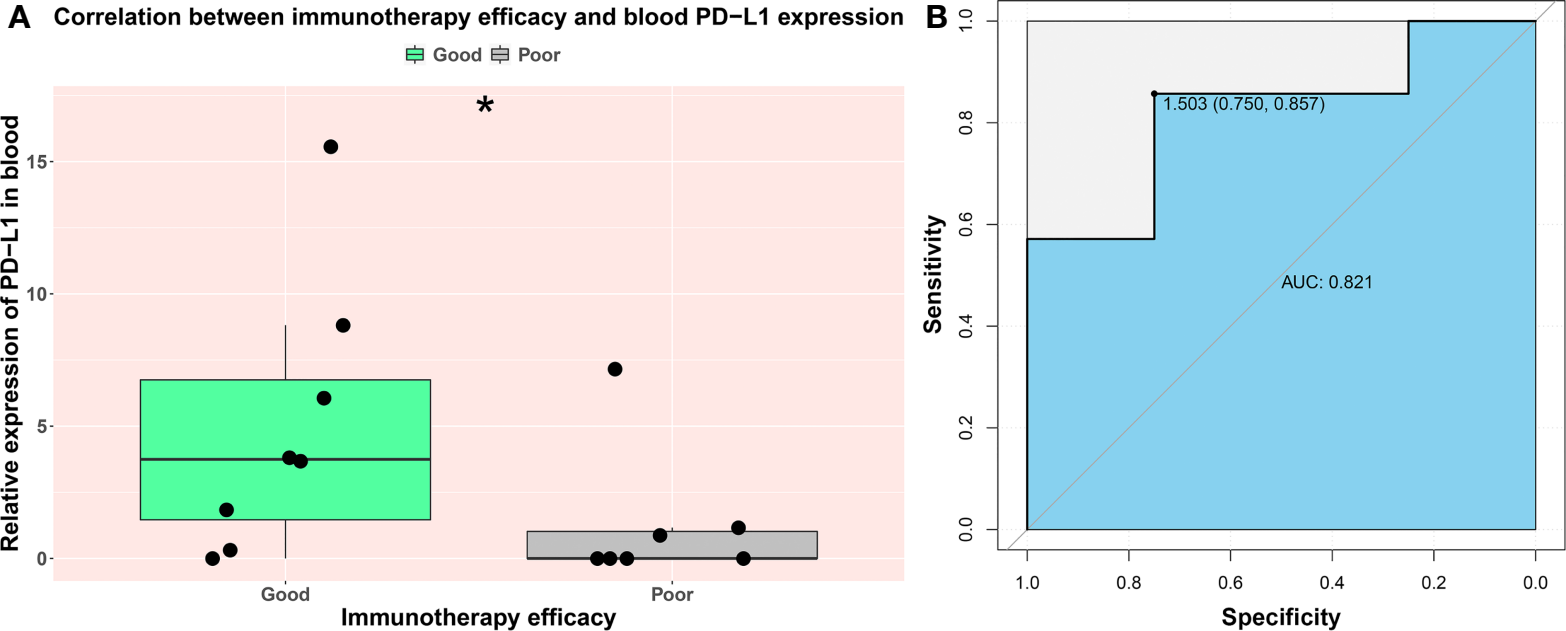
The prognostic value of PD-L1 expression in blood on immune efficacy. Each patient was grouped into a good (CR and PR) and poor (SD and PD) cohort based on their immunotherapy outcomes. **(A)** The comparison of blood PD-L1 expression level between good and poor cohort. **(B)** The ROC curve of blood PD-L1expression and immunotherapy efficacy. AUC indicates area under the ROC curve; “*” indicates *p-value* < 0.05.

A small cohort of prospective study was designed for discovering the potential ability of blood PD-L1 mRNA expression level in predicting immunotherapy efficacy. A total of 15 patients with STAD were enrolled, and three of them received surgery before immunotherapy. Blood samples of each patient were obtained pre- immunotherapy, and PD-L1 mRNA expression was quantified as the method described before. Patients were grouped into good [including complete response (CR) and partial response (PR)] and poor [including stable disease (SD) and progressive disease (PD)] cohorts building upon their treatment effectiveness. Followed by three-course immunotherapy, eight patients were evaluated as good responders, and others were classified as poor responders (**Supplementary Table S6**). Our results showed that, compared with the poor cohort, PD-L1 expression in blood was significantly higher in the good cohort (*p-value* = 0.04, **Figure 5A**).

Moreover, IHC assays were performed on the tissue specimens taken during the surgical removal of tumors of the four patients. Three patients’ assessment results are ≥1, and their paired blood PD-L1 mRNA expression levels (*2^−ΔΔct^ value* = 6.1, 1.8, and 15.6, respectively) were significantly higher than another patient whose assessment result is <1 (*2^−ΔΔct^ value* = 7.8e^−6^). Notably, consistent with our prospection, all of these three patients with high expression maintained clinical benefits from immunotherapy, whereas another patient with low expression was verified as a poor responder (**Supplementary Table S6**).

### The cutoff value of blood PD-L1 mRNA expression in predicting immunotherapy efficacy

We further investigated the clinical application value of the blood PD-L1 mRNA test. The ROC curve was drawn to analyze the optimal threshold. Our data show that the area under the ROC curve (AUC) was 0.821, and the best cutoff of relative expression level is *2^−ΔΔct^ value* = 1.503, which balanced the specificity (75.0%) and sensitivity (85.7%) (**Figure 5B**). Our results indicated that the blood PD-L1 mRNA expression level is a powerful potential marker for immunotherapy efficacy of patients with STAD.

## Discussion

Previous studies explored the relationship between the PD-L1 expression and the baseline information of the patients, risk of morbidity, and prognosis. Overall, the study evidence did not support an association between PD-L1 expression and patient baseline information such as age, gender, and tumor stage in lung cancer (8), but higher expression levels of PD-L1 were associated with poor prognosis in lung cancer (23) and melanoma (24). However, followed by a large sample size survey in TCGA database, our study shows that the PD-L1 expression is less correlated to the survival of patients with gastrointestinal cancer but significantly related to the tumor stage of patients with COAD, as well as the primary therapy outcomes of patients with READ. This result indicates the potential application of PD-L1 expression level in the stratification of patients with gastrointestinal tumor.

Theoretically speaking, subjective judgment is essential for IHC diagnosis, which is far influenced by the personal experience of the pathologist. Compared with the IHC test, qRT-PCR experiment is an objective and reliable semi-quantitative approach that observer-independent evaluated, which has the advantage of simple, accurate, easy to use, and low cost in time and money (25). This study indicates a numerically but not statistically significant consistency of PD-L1 expression level between IHC assay and qRT-PCR test and explored the potential technical feasibility of using qRT-PCR to replace IHC in PD-L1 expression detection of gastrointestinal tumors.

Some studies could support our findings, for instance, Xiao and colleagues assessed the expression levels of mRNA and protein of PD-L1 in 330 patients with non-metastatic clear cell renal cell carcinoma *via* qRT-PCR and IHC test, respectively, and 292 patients had consistent results for mRNA and the PD-L1 protein (26), indicating that the evaluation of PD-L1 expression based on qRT-PCR technology is predictive valuable. Another study showed that, depending on the 28-8 antibody employed, the agreement between PD-L1 mRNA and IHC staining in primary non-small cell lung cancer tissues was excellent, and the differences became apparent in metastatic lesions (27). However, these conclusions remain controversial. Some researchers compared the PD-L1 expression in tumor tissue and circulating tumor cells, and they demonstrated that PD-L1 expression did not correspond completely (28).

Notably, PD-L1 mRNA expression level may also be advantageous to the clinical medication guidance. For instance, PD-L1 mRNA expression in platelet and exosomes also contribute to predicting the clinical response to immunotherapy (29), the measurement of PD-L1 mRNA expression in tumor tissue by RNA-seq is analytically and clinically in predicting the immune checkpoint inhibitor response (30), and blood expressed PD-L1 mRNA level was confirmed to be associated with anti–PD-1 response (12–14). Moreover, compared with the healthy controls, tissue PD-L1 mRNA expression decreased in patients with colorectal cancer (31) and could successfully discriminate the worse progression-free survival of those patients with colorectal cancer liver metastases who adopted neoadjuvant-treatment (32). However, some literature studies express the opposite view and reported that no statistically significant difference in the PD-L1 mRNA expression between responders and non-responders to immunotherapy was observed (33). In addition, the prognostic value of PD-L1 also remains controversial. A review of 15 published studies in patients with GC revealed that PD-L1 expression was a negative prognostic factor for OS in 11 studies and a positive prognostic factor in three studies, with one study reporting no prognostic significance (34).

We hypothesized that the different types of specimens and measurement methods may contribute to this controversy. Under the strictly identical process and manual operation, the mRNA expression of PD-L1 was quantified in tumor tissue samples obtained from the 52 enrolled patients by the qRT-PCR method. Our results suggested that no significant relationship between the tumor cell content and the PD-L1 expression level was observed in tumor tissue specimens, and this may be attributed to the extra source of PD-L1 mRNA obtained from non-tumor cells. However, compared with the low- expression (<1%) cohort quantitated by IHC assay, the average expression level of PD-L1 mRNA is higher in the high- expression (>1%) cohort. Interestingly, similar results were denoted in the paired blood samples, which may establish a liquid-biopsy approach that could provide a broad range of opportunities in the field of oncology and clinically benefit patients unable to obtain tissue specimens (35). Hence, we inquired about the correlation between blood-derived PD-L1 expression and immunotherapy efficacy. As expected, results verified that patients with GC with high PD-L1 expression (relative expression level over 1.5) have a better chance to harvest a good response to immunotherapy (*p-value* = 0.04) and to achieve a specificity rate of 75.0% and a sensitivity rate of 85.7%.

However, this study has several limitations. First, this is a single-center, small sample-size study. Second, this study did not investigate whether the tumor stage is related to the PD-L1 expression level. Third, recently, a complex co-existence between microsatellite instability (MSI), tumor mutation burden (TMB), and PD-L1 expression in the therapeutic effect of immunotherapy was discovered (36); however, this study did not investigate the MSI and TMB state. Fourth, the correlation between blood PD-L1 expression and immunotherapy efficacy of patients with COAD and READ remains unclear. Therefore, to expand the understanding of the predicting ability of qRT-PCR–based blood PD-L1 expression test in response to immunotherapeutic drugs of patients with gastrointestinal cancer, multicenter studies involving a larger cohort and comprehensive analysis involving MSI and TMB should be further developed.

## Conclusion

In conclusion, we compared and reported the potential consistency between IHC and qRT-PCR results of PD-L1 expression detection in patients with gastrointestinal cancer. We found that the qRT-PCR detection performed in both blood and tissue specimens was numerically but not statistically significant in accordance with IHC assay (corresponding to a *p-value* of 0.84 and 0.55, respectively), and PD-L1 expression level in blood could independently predict the efficacy of immunotherapy in patients with GC (*p-value* = 0.04). Moreover, we discovered the PD-L1 relative expression cutoff value of good immune efficacy is 1.5, in which the specificity and sensitivity rates of prediction were 75.0%, and 85.7%, respectively. Our study established a novel strategy for rapidly well-identified patients with GC with the response to immunotherapy and has potential clinical benefits.

## Data availability statement

The original contributions presented in the study are included in the article/**Supplementary Material**. Further inquiries can be directed to the corresponding authors.

## Ethics statement

The studies involving human participants were reviewed and approved by Internal Review Board of the Ruijin Hospital Affiliated to Shanghai Jiaotong University School of Medicine (IRB approval No. LWEC2020010). The patients/participants provided their written informed consent to participate in this study.

## Author contributions

The experiments were designed by MW, DZ, and ZG. MW, DZ, and QQ were involved in project administration. MW, QQ, DZ, ZG, JL, and XZ performed experiments and analyzed the data. The first draft of the manuscript was written by JL and XZ. JL performed data visualization and reviewed and edited the manuscript. All authors have read and approved the final manuscript.

## Funding

This work was supported by the Shanghai medicine key specialty (ZK2019B17), the Training plan for outstanding young professionals (YQA202006), the Healthy Commission Research Project of Shanghai Huangpu District (HLM202001), the Zhejiang Leading Talent Entrepreneurship Project (2021R02019), the Jiaxing Leading Talent Entrepreneurship Project, and the Key Technology Innovation Projects of Jiaxing (2021BZ10004).

## Conflict of interest

Author JL is employed by the Zhejiang Yunying Medical Technology Co., Ltd. and the Jiaxing Yunying Medical Inspection Co., Ltd.; author XZ is employed by the Zhejiang Yunying Medical Technology Co., Ltd. and the Jiaxing Yunying Medical Inspection Co., Ltd.; author ZG is employed by the Zhejiang Yunying Medical Technology Co., Ltd. and the Jiaxing Yunying Medical Inspection Co., Ltd.; author DZ is employed by the Zhejiang Yunying Medical Technology Co., Ltd. and the Jiaxing Yunying Medical Inspection Co., Ltd.

The remaining authors declare that the research was conducted in the absence of any commercial or financial relationships that could be construed as a potential conflict of interest.

## Publisher’s note

All claims expressed in this article are solely those of the authors and do not necessarily represent those of their affiliated organizations, or those of the publisher, the editors and the reviewers. Any product that may be evaluated in this article, or claim that may be made by its manufacturer, is not guaranteed or endorsed by the publisher.
