# Supplementary material for: Assessment of PD-L1 mRNA expression in gastrointestinal tumors and the response to immunotherapy
Source: Front Oncol. 2022 Dec 1;12:926746. doi: 10.3389/fonc.2022.926746 (PMC9751311; doi:10.3389/fonc.2022.926746)
Supplement: Supplementary file 1 [file DataSheet_1.pdf]

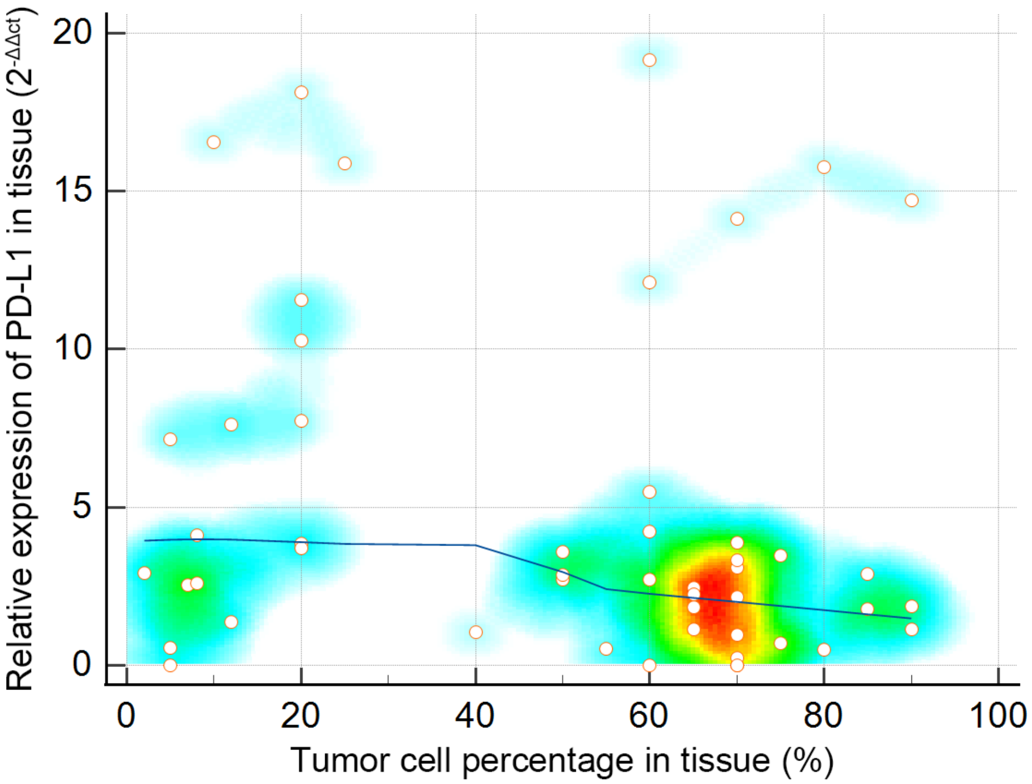

Supplementary Figure S1. Correlation between the percentage of tumor cells and the mRNA expression of PD-L1. Pearson's correlation coefficient test is used in statistical analysis, each dot indicates a sample, and the blue line represents the fitting curve.

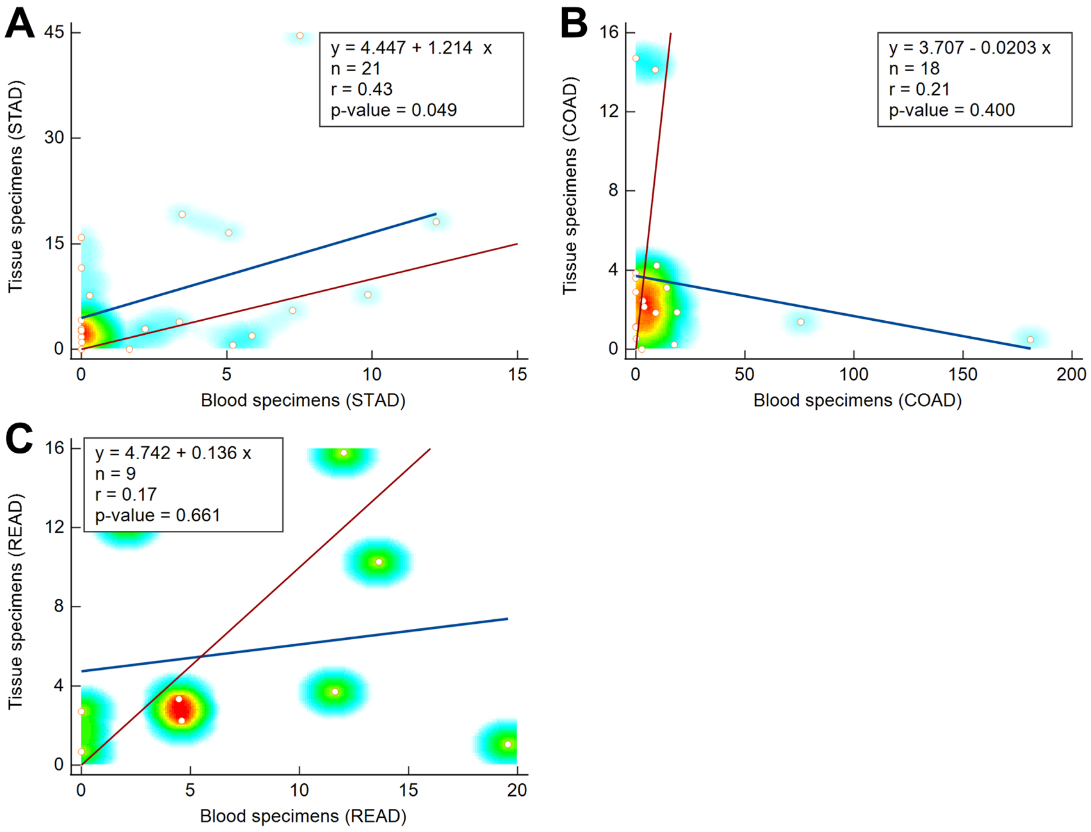

Supplementary Figure S2. Linear regression analysis of PD-L1 mRNA expression level in blood specimens and tissue specimens. A), B), and C) indicates the STAD patients, COAD patients, and READ patients, respectively. Student's T test is used in statistical analysis, each dot indicates a sample, the blue line represents the fitting curve, and the red line indicates the line of equality (y = x).

Supplementary Table S1. Primers and probes used for qRT-PCR expression analysis.

	Forward primer sequence (5'-3')	Reverse primer sequence (5'-3')	TaqMan probe sequence (5'-3')
PD-L1	AGCACTGACATTCATCTTCCG	GCTGGATTACGTCTCCTCCA	AATGTGGCATCCAAGATAC
GAPDH	CTCTGCTCCTCCTGTTCGAC	ATGGTGTCTGAGCGATGTGG	CGTCGCCAGCCGA

Supplementary Table S2. Detailed information of 11 normal controls.

Sample ID	Gender	Age	Relative expression in blood specimens			
			PD-L1$_{(Ct)}$	GAPDH$_{(Ct)}$	Δct	Average Δct
N1	Female	29	18.91	4.82	14.09	
N2	Male	22	17.96	6.05	11.91	
N3	Female	25	20.36	7.57	12.79	
N4	Female	28	21.62	7.01	14.61	
N5	Female	25	21.46	7.28	14.18	
N6	Male	32	21.04	8.59	12.45	13.01
N7	Female	28	20.23	7.64	12.59	
N8	Male	28	20.58	7.91	12.67	
N9	Female	32	20.18	7.61	12.57	
N10	Female	28	20.43	7.10	13.33	
N11	Female	27	20.13	8.21	11.92	

Supplementary Table S3. Detailed information of patients.

Patient ID	Age at Diagnosis in Years	Gender	Clinical diagnosis	Tumor size and cancer stage		
				Size (length*width, cm)	TNM staging system	Stage
1	57	Male	READ	3.5*2.5	$T_{4a}N_{1c}M_0$	IIIB
2	69	Female	COAD	5*4	$T_{4a}N_{2a}M_0$	IIIC
3	61	Male	STAD	1.5*1.5	$T_2N_2M_0$	IIB
4	67	Female	READ	4*3	$T_2N_0M_0$	I
5	69	Male	COAD	2.5*2.5	$T_{4a}N_1M_0$	IIIB
6	53	Female	COAD	5	$T_{4a}N_{2b}M_0$	IIIC
7	70	Male	COAD	4.5*2	$T_2N_0M_0$	I
8	63	Female	COAD	4*2.5	$T_3N_2M_0$	IIIC
9	61	Male	READ	5	$T_{4a}N_0M_0$	IIB
10	63	Female	STAD	3*3	$T_3N_1M_0$	IIB
11	68	Male	READ	3.5	$T_{4a}N_2M_0$	IIIC
12	83	Female	COAD	12*10	$T_{4b}N_{1c}M_0$	IIIB
13	59	Female	STAD	2.5*2	$T_{1b}N_3M_0$	IIB
14	68	Male	STAD	2.5*2	$T_2N_0M_0$	IB
15	73	Male	STAD	4*3	$T_{4a}N_3M_0$	IIIC
16	50	Female	READ	1.5*1.5	$T_{1b}N_0M_0$	I
17	64	Male	STAD	3.5*3	$T_2N_0M_0$	IB
18	53	Female	STAD	1.5*1	$T_{1a}N_0M_0$	IA
19	49	Female	READ	5*3.5	$T_{4b}N_1M_0$	IIIB
20	64	Male	COAD	2.5*2.5	$T_2N_{1c}M_0$	IIIA
21	60	Male	COAD	2*2	$T_{4a}N_1M_0$	IIIB
22	76	Male	STAD	5*4.5	$T_{4a}N_3M_0$	IIIC
23	57	Female	STAD	3*2	$T_2N_1M_0$	IIA
24	77	Male	STAD	5*4	$T_2N_0M_0$	IB
25	42	Male	COAD	6*10	$T_{4b}N_0M_0$	IIC
26	78	Male	COAD	4*2.5	$T_{4a}N_1M_1$	IV
27	56	Female	STAD	1*1	$T_{1a}N_0M_0$	IA
28	34	Female	COAD	1	$T_2N_0M_0$	I
29	77	Male	STAD	5.5*4.5	$T_{4b}N_2M_0$	IIIC
30	71	Male	STAD	3*2.5	$T_2N_2M_0$	IIB
31	63	Male	READ	4*3	$T_{4a}N_0M_0$	IIB
32	42	Female	COAD	2.5*2*1	$T_{4a}N_2M_1$	IV
33	35	Female	COAD	6*8	$T_4N_0M_0$	IIB
34	58	Male	COAD	1.5*1.5	$T_{4a}N_{1c}M_1$	IV
35	65	Male	STAD	2*1.5	$T_{1b}N_0M_0$	IA
36	60	Female	STAD	5*4.5*2	$T_{4a}N_3M_0$	IIIC
37	76	Female	STAD	5*5	$T_3N_0M_0$	IIA
38	65	Male	STAD	4*2	$T_{4a}N_2M_0$	IIIB
39	66	Male	READ	3.5*3	$T_2N_1M_0$	IIIA
40	60	Male	COAD	8*10	$T_4N_2M_1$	IV
41	77	Female	COAD	4.5*4	$T_4N_1M_0$	IIIA
42	60	Male	READ	3.5*3	$T_2N_2M_0$	IIIB
43	71	Male	COAD	2.5*10	$T_4N_1M_0$	IIIB
44	81	Male	READ	2.5*2	$T_{4a}N_0M_0$	IIB
45	66	Male	STAD	6*3.5	$T_{4a}N_1M_0$	IIIA
46	52	Female	STAD	6*5	$T_{yp4a}N_{yp0}M_1$	IV
47	66	Female	COAD	2*2	$T_2N_0M_0$	IB
48	67	Male	STAD	4.5*3.5	$T_{4a}N_3M_0$	IIIC
49	70	Male	STAD	2.5*2	$T_{4b}N_0M_1$	IV
50	65	Female	STAD	3.5*3	$T_{yp1b}N_{yp1}M_0$	ypII
51	65	Female	STAD	2*1.5	$T_2N_3M_0$	IIIA
52	46	Male	COAD	3.5*3	$T_2N_0M_0$	I

Note:

Abbr. STAD, stomach adenocarcinoma; COAD, colon adenocarcinoma; READ, rectum adenocarcinoma.

Supplementary Table S4. The detection results of PD-L1 expression.

Patient ID	IHC result of PD-L1	Tumor cell percentage$_{(\%)}$	Relative expression in blood specimens			Relative expression in tissue specimens		
			**PD-L1$_{(Ct)}$**	**GAPDH$_{(Ct)}$**	**$2^{-\Delta\Delta Ct}$**	**PD-L1$_{(Ct)}$**	**GAPDH$_{(Ct)}$**	**$2^{-\Delta\Delta Ct}$**
1	<1%	70	17.96	7.11	4.47	22.12	10.85	3.34
2	<1%	65	35	8.39	$8.05e^{-5}$	22.95	10.11	1.13
3	<1%	12	23.76	8.96	0.29	20.53	10.45	7.62
4	<1%	75	35	8.16	$6.87e^{-5}$	20.96	7.41	0.69
5	<1%	60	18.27	6.76	2.83	35	12.17	$1.11e^{-3}$
6	<1%	70	18.28	7.19	3.78	22.11	10.21	2.16
7	<1%	65	18.96	7.58	3.10	21.86	10.14	2.45
8	<1%	65	16.09	6.27	9.13	22.15	10.03	1.85
9	<1%	60	19.48	7.52	2.07	19.39	9.98	12.13
10	>1%	10	18.68	8.01	5.06	20.11	11.15	16.56
11	<1%	65	17.86	7.05	4.59	21.89	10.05	2.25
12	<1%	85	35	8.29	$7.51e^{-5}$	19.49	8.01	2.89
13	>1%	8	35	10.93	$4.68e^{-4}$	21.44	10.47	4.11
14	>1%	50	35	10.97	$4.82e^{-4}$	21.82	10.25	2.71
15	>1%	70	19.33	8.07	3.36	20.79	9.74	3.89
16	<1%	20	17.55	8.08	11.63	21.7	10.58	3.71
17	>1%	5	18.51	7.88	5.21	24.24	10.39	0.56
18	<1%	2	19.95	8.07	2.19	23.72	12.25	2.91
19	<1%	40	16.75	8.03	19.56	21.42	8.47	1.04
20	>1%	12	16.76	9.99	75.58[*]	21.35	8.8	1.38
21	>1%	70	17.09	8.22	17.63	25.45	10.34	0.23
22	>1%	85	35	9.34	$1.56e^{-4}$	21.92	9.74	1.78
23	/	/	18.35	7.89	5.86	23.99	11.89	1.88
24	>1%	70	27.15	8.57	0.02	21.6	8.53	0.96
25	<1%	80	14.16	8.65	181.02[*]	22.53	8.47	0.48
26	<1%	70	17.75	8.56	14.12	20.82	9.44	3.10
27	<1%	7	35	9.43	$1.66e^{-4}$	19.96	8.29	2.53
28	<1%	90	40	12.19	$3.51e^{-5}$	22.86	10.03	1.13
29	<1%	5	16.98	4.69	1.65	40	8.24	$2.27e^{-6}$
30	<1%	8	/	/	/	25.91	14.28	2.60
31	<1%	20	21.36	12.12	13.64	21.63	11.98	10.27
32	<1%	55	21.57	5.91	0.16	22.87	8.94	0.53
33	>1%	60	14.74	4.98	9.51	21.66	10.73	4.23
34	<1%	20	40	9.33	$4.83e^{-6}$	17.83	6.77	3.86
35	>1%	25	40	8.51	$2.74e^{-6}$	17.74	8.72	15.89
36	>1%	5	/	/	/	16.81	6.64	7.16
37	<1%	20	40	8.45	$2.62e^{-6}$	19.46	9.98	11.55
38	>1%	20	19.74	10.03	9.85	18.04	7.98	7.73
39	>1%	75	/	/	/	18.44	7.23	3.48
40	<1%	70	/	/	/	35	14.02	$3.99e^{-3}$
41	>1%	90	40	6.64	$7.48e^{-7}$	14.01	4.88	14.72
42	<1%	80	19.97	10.55	12.04	16.88	7.85	15.78
43	<1%	90	18.75	9.98	18.90	18.06	5.96	1.88
44	<1%	60	40	12.48	$4.29e^{-5}$	17.89	6.32	2.71
45	<1%	60	18.54	7.32	3.46	16.62	7.87	19.16
46	<1%	20	18.95	9.55	12.21	17.96	9.13	18.13
47	<1%	70	18.74	8.89	8.94	16.02	6.83	14.12
48	<1%	60	19.03	8.88	7.26	19.22	8.67	5.50
49	<1%	50	40	10.12	$8.35e^{-6}$	19.57	8.08	2.87
50	>1%	95	19.33	9.23	7.52	18.09	10.56	44.63[*]
51	<1%	70	40	10.88	$1.41e^{-5}$	35	11.88	$9.05e^{-4}$
52	<1%	50	40	9.97	$7.52e^{-6}$	18.92	7.76	3.61

Note:

[*], the extreme values were removed in the further analysis.

Supplementary Table S5. Statistical analysis of PD-L1 expression level between protein and mRNA.

	**Average relative expression of PD-L1 mRNA (mean ± standard deviation)**		**F value**	**p-value**
Protein level	<1%	>1%		
Total				
Blood specimen	4.88 ± 5.88[*]	4.47 ± 5.25[*]	0.77	0.84
Tissue specimen	4.68 ± 5.31	5.69 ± 5.44[*]	0.79	0.55
STAD				
Blood specimen	2.71 ± 3.85	3.10 ± 3.48	0.77	0.82
Tissue specimen	6.62 ± 6.50	6.14 ± 5.52[*]	0.65	0.86
COAD				
Blood specimen	4.69 ± 5.99[*]	9.05 ± 7.21[*]	0.35	0.32
Tissue specimen	2.61 ± 3.30	5.14 ± 5.72	0.07	0.30
READ				
Blood specimen	7.56 ± 6.50			
Tissue specimen	5.77 ± 5.17	3.48 ± 0.00[#]		

Note:

[*], removed one extreme value; [#], only one case was included; *Abbr.* STAD, stomach adenocarcinoma; COAD, colon adenocarcinoma; READ, rectum adenocarcinoma.

Supplementary Table S6. Detailed information of patients who received immunotherapy.

Patient ID	Age at Diagnosis in Years	Gender	Clinical diagnosis	Tumor size and cancer stage			IHC result of PD-L1	Relative expression of PD-L1 in blood			Immune efficacy
				Size (length*width, cm)	TNM staging system	Stage		PD-L1$_{(Ct)}$	GAPDH$_{(Ct)}$	$2^{-\Delta\Delta Ct}$	
T1	57	Female	STAD	/	$T_XN_XM_1$	IV	/	35	9.11	$1.33e^{-4}$	SD (Poor)
T2	60	Male	STAD	3.5*2	$T_4N_2M_1$	IV	5-7	19.93	9.52	6.06	PR (Good)
T3	81	Male	STAD	/	$T_XN_XM_1$	IV	/	40	10.75	$1.29e^{-5}$	SD (Poor)
T4	38	Male	STAD	6*5*2	$T_4N_3M_1$	IV	/	19.13	8.96	7.16	PD (Poor)
T5	60	Female	STAD	5*4.5*2	$T_4N_3M_0$	III	<1	40	10.02	$7.79e^{-6}$	PD (Poor)
T6	49	Male	STAD	4*3*1	$T_4N_3M_0$	III	1	18.34	6.21	1.84	PR (Good)
T7	69	Male	STAD	/	$T_XN_XM_1$	IV	/	20.98	6.33	0.32	PR (Good)
T8	65	Female	STAD	/	$T_XN_XM_1$	IV	/	35	9.13	$1.35e^{-4}$	PR (Good)
T9	65	Female	STAD	8*6*2	$T_4N_3M_0$	III	/	40	5.85	$4.33e^{-7}$	SD (Poor)
T10	38	Male	STAD	4.5*4*1.8	$T_4N_2M_1$	IV	10	17.75	8.7	15.56	PR (Good)
T11	66	Female	STAD	/	$T_XN_XM_1$	IV	/	20.09	6.89	0.88	SD (Poor)
T12	54	Male	STAD	7*5*1	$T_4N_3M_1$	IV	/	16.98	5.9	3.81	PR (Good)
T13	63	Male	STAD	/	$T_XN_XM_1$	IV	/	20.81	8.02	1.16	SD (Poor)
T14	63	Female	STAD	/	$T_4N_2M_1$	IV	/	18.55	8.68	8.82	PR (Good)
T15	49	Male	STAD	8*5*3	$T_4N_3M_0$	III	/	20.02	8.89	3.68	PR (Good)

Note:

Abbr. STAD, stomach adenocarcinoma; SD, stable disease; PD, progressive disease; PR, partial response.
